# {2-[1-(2-Amino-2-methyl­propyl­imino)eth­yl]phenolato-κ^3^ *N*,*N*′,*O*}dioxidovanadium(V)

**DOI:** 10.1107/S1600536808042839

**Published:** 2009-01-14

**Authors:** Grzegorz Romanowski, Michał Wera, Artur Sikorski

**Affiliations:** a University of Gdańsk, Faculty of Chemistry, Sobieskiego 18/19, 80-952 Gdańsk, Poland

## Abstract

In the crystal structure of the title compound, [V(C_12_H_17_N_2_O)O_2_], the vanadium(V) centre is five-coordinate in a distorted square-pyramidal environment. The three atoms of the deprotonated Schiff base and a double-bonded O atom comprise the basal plane. N—H⋯O hydrogen bonds lead to a zigzag chain structure parallel to [001].

## Related literature

For general background, see: Carter-Franklin *et al.* (2003 ▶); Eady (2003 ▶); Evangelou (2002 ▶); Mendz (1991 ▶); Mokry & Carrano (1993 ▶); Parekh *et al.* (2006 ▶); Rehder *et al.* (2002 ▶, 2003 ▶); Shahzadi *et al.* (2007 ▶). For related structures, see: Kwiatkowski *et al.* (2003 ▶, 2007 ▶); Rao *et al.* (1981 ▶). For synthesis, see: Kwiatkowski *et al.* (2003 ▶). For the calculation of square-pyramidal geometries, see: Holmes (1984 ▶).
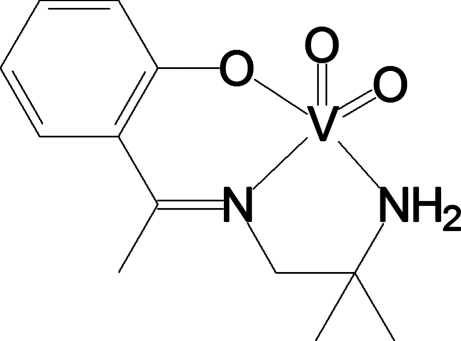


## Experimental

### 

#### Crystal data

[V(C_12_H_17_N_2_O)O_2_]
*M* *_r_* = 288.22
Orthorhombic, 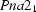

*a* = 11.1198 (6) Å
*b* = 15.7408 (8) Å
*c* = 7.6448 (3) Å
*V* = 1338.10 (11) Å^3^
*Z* = 4
Mo *K*α radiation
μ = 0.74 mm^−1^
*T* = 295 (2) K
0.20 × 0.04 × 0.04 mm

#### Data collection

Oxford Diffraction Ruby CCD diffractometer
Absorption correction: multi-scan (*CrysAlis RED*; Oxford Diffraction, 2008 ▶) *T*_min_ = 0.941, *T*_max_ = 0.964
11574 measured reflections
2126 independent reflections
1387 reflections with *I* > 2σ(*I*)
*R*_int_ = 0.071

#### Refinement

*R*[*F* ^2^ > 2σ(*F* ^2^)] = 0.029
*wR*(*F* ^2^) = 0.051
*S* = 0.83
2126 reflections
167 parameters
1 restraint
H-atom parameters constrained
Δρ_max_ = 0.19 e Å^−3^
Δρ_min_ = −0.16 e Å^−3^
Absolute structure: Flack (1983 ▶), 849 Friedel pairs
Flack parameter: 0.23 (2)

### 

Data collection: *CrysAlis CCD* (Oxford Diffraction, 2008 ▶); cell refinement: *CrysAlis RED* (Oxford Diffraction, 2008 ▶); data reduction: *CrysAlis RED*; program(s) used to solve structure: *SHELXS97* (Sheldrick, 2008 ▶); program(s) used to refine structure: *SHELXL97* (Sheldrick, 2008 ▶); molecular graphics: *ORTEPII* (Johnson, 1976 ▶); software used to prepare material for publication: *SHELXL97* and *PLATON* (Spek, 2003 ▶).

## Supplementary Material

Crystal structure: contains datablocks I, global. DOI: 10.1107/S1600536808042839/ng2526sup1.cif

Structure factors: contains datablocks I. DOI: 10.1107/S1600536808042839/ng2526Isup2.hkl

Additional supplementary materials:  crystallographic information; 3D view; checkCIF report

## Figures and Tables

**Table 1:** Hydrogen-bond geometry (Å, °)

*D*—H⋯*A*	*D*—H	H⋯*A*	*D*⋯*A*	*D*—H⋯*A*
N11—H11*A*⋯O14^i^	0.90	2.08	2.942 (4)	159
N11—H11*B*⋯O15^ii^	0.90	2.29	3.173 (4)	168

Symmetry codes: (i) 
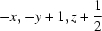
; (ii) 
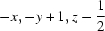
.

**Fig. 1.**
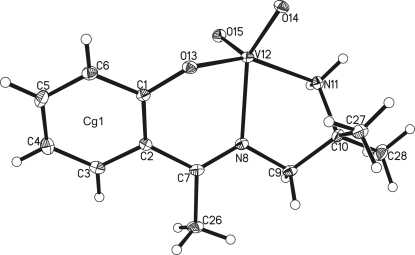
The molecular structure of the title complex (I), with displacement ellipsoids drawn at the 25% probability level and H atoms are shown as small spheres of arbitrary radius.

**Fig. 2.**
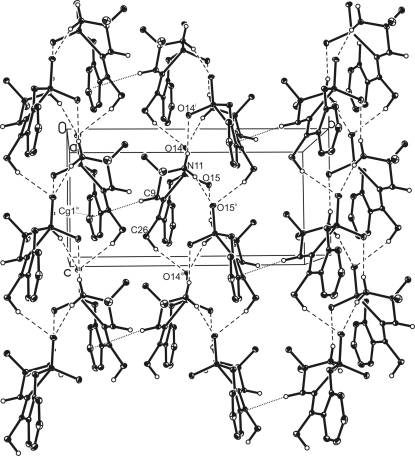
The arrangement of the molecules of (I) in the crystal structure viewed approximately along a axis. The C—H···O and N—H···O hydrogen bonds are represented by dashed lines and the C—H···π interactions are represented by dotted lines.

## supplementary crystallographic information

### Comment

Vanadium(IV) and (V) complexes with Schiff bases are excellent model compounds for some biological enzymes, *viz*.: haloperoxidases (Carter-Franklin *et al.*, 2003; Rehder *et al.*, 2003), phosphomutases (Mendz, 1991) and nitrogenases (Eady, 2003). Their various pharmocological properties also has been reported, especially as an antidiabetes (Rehder *et al.*, 2002), anticancer (Evangelou, 2002), antifungal and antibacterial (Parekh *et al.*, 2006; Shahzadi *et al.*, 2007) agents.

The crystal structure of (I) consists of monomeric complex molecules (Fig. 1) with a distorted square pyramidal geometry about vanadium with O15 at the apex. By the use of the dihedral angle method and the unit bond lengths, it was estimated that the structure is displaced by 76.1% along the Berry coordinate from the ideal trigonal bipyramidal (0%) toward the ideal square pyramidal (100%) geometry (Holmes, 1984). In contrast to this structure, the similar monomeric complex, but derived from 4,6-dimetoxysalicylaldehyde (Kwiatkowski *et al.*, 2003), reveals a distorted trigonal bipyramidal environment with the degree of the distortion of 41.2% along the Berry coordinate. The vanadium atom is displaced from the mean plane passing through the four basal atoms, N8, N11, O13 and O14, by *ca* 0.51 (1) Å towards O15. The bond lengths V12—N8 of 2.171 (3) Å, V12—N11 of 2.118 (2) Å, V12—O13 of 1.883 (2) Å, V12—O14 of 1.635 (2) Å, V12—O15 of 1.615 (2) Å and the O=V=O angle of 109.9° are similar to the values in other *cis*-VO_2_^+^ complexes (Mokry & Carrano, 1993; Kwiatkowski *et al.*, 2003; Kwiatkowski *et al.*, 2007). The six-membered chelate ring (V12, O13, C1, C2, C7, N8) is concluded to be an envelope on V12 atom. The ring puckering analysis shows that the five-membered chelate ring defined by V12, N11, C10, C9, N8 atoms adopts a twisted conformation on C10 and N11 atoms, with P = 282.7 (2)° and Tau_(_*_M_*_)_ = 49.9 (2)° for reference bond V12—N8 (Rao *et al.*, 1981).

The crystal structure of the monomeric complex (I) is stabilized by the C—H···O, N—H···O hydrogen bonds and C—H···π interactions. Hydrogen bonds between dioxidovanadium oxygen atoms and nitrogen and carbon atoms of neighbouring molecules result in formation of infinite chains and closed loops, extending in the *a* direction (Tables 1 and 2, Fig. 2).

### Experimental

The complex (I) were obtained in a template/complexation reactions analogous to those described for preparation of dioxidovanadium(V) complexes with Schiff base ligands (Kwiatkowski *et al.*, 2003). A solution of 1 mmol of 2-methyl-1,2-diaminopropane in 10 ml of absolute ethanol was added under stirring to a freshly filtered solution of vanadium(V) oxytriethoxide (1 mmol) in 50 ml of absolute EtOH producing a yellow suspension of the intermediate. 2-Acetylphenol (1 mmol) dissolved in absolute EtOH was added to the aforementioned suspension. After refluxing (70 ml) of the resulting mixture for 2 h and its cooling to room temperature the separated solids were filtered off, washed several times with EtOH and dried over molecular sieves.

### Refinement

All H atoms were positioned geometrically and refined using a riding model, with C–H distances of 0.93–0.97Å and with *U*_iso_(H) = 1.2*U*_eq_(C) (C–H = 0.96Å and *U*_iso_(H) = 1.5*U*_eq_(C) for the methyl group) and with N–H distances of 0.90Å and with *U*_iso_(H) = 1.2*U*_eq_(C).

### Crystal data

**Table:**

[V(C_12_H_17_N_2_O)O_2_]	*F*(000) = 600
*M**_r_* = 288.22	*D*_x_ = 1.431 Mg m^−^^3^
Orthorhombic, *P**n**a*2_1_	Mo *K*α radiation, λ = 0.71073 Å
Hall symbol: P 2c -2n	Cell parameters from 2126 reflections
*a* = 11.1198 (6) Å	θ = 3.2–25.1°
*b* = 15.7408 (8) Å	µ = 0.74 mm^−^^1^
*c* = 7.6448 (3) Å	*T* = 295 K
*V* = 1338.10 (11) Å^3^	Needle, white
*Z* = 4	0.2 × 0.04 × 0.04 mm

### Data collection

**Table:**

Oxford Diffraction Ruby CCD diffractometer	2126 independent reflections
Radiation source: Enhance (Mo) X-ray Source	1387 reflections with *I* > 2σ(*I*)
graphite	*R*_int_ = 0.071
Detector resolution: 10.4002 pixels mm^-1^	θ_max_ = 25.1°, θ_min_ = 3.2°
ω scans	*h* = −12→13
Absorption correction: multi-scan (*CrysAlis RED*; Oxford Diffraction, 2008)	*k* = −18→18
*T*_min_ = 0.941, *T*_max_ = 0.964	*l* = −9→7
11574 measured reflections	

### Refinement

**Table:**

Refinement on *F*^2^	Secondary atom site location: difference Fourier map
Least-squares matrix: full	Hydrogen site location: inferred from neighbouring sites
*R*[*F*^2^ > 2σ(*F*^2^)] = 0.029	H-atom parameters constrained
*wR*(*F*^2^) = 0.051	*w* = 1/[σ^2^(*F*_o_^2^) + (0.0211*P*)^2^] where *P* = (*F*_o_^2^ + 2*F*_c_^2^)/3
*S* = 0.83	(Δ/σ)_max_ < 0.001
2126 reflections	Δρ_max_ = 0.19 e Å^−^^3^
167 parameters	Δρ_min_ = −0.16 e Å^−^^3^
1 restraint	Absolute structure: Flack (1983), 849 Friedel pairs
Primary atom site location: structure-invariant direct methods	Flack parameter: 0.23 (2)

### Special details

**Table:**

Geometry. All e.s.d.'s (except the e.s.d. in the dihedral angle between two l.s. planes)are estimated using the full covariance matrix. The cell e.s.d.'s are takeninto account individually in the estimation of e.s.d.'s in distances, anglesand torsion angles; correlations between e.s.d.'s in cell parameters are onlyused when they are defined by crystal symmetry. An approximate (isotropic)treatment of cell e.s.d.'s is used for estimating e.s.d.'s involving l.s. planes.
Refinement. Refinement of *F*^2^ against ALL reflections. The weighted *R*-factor *wR* andgoodness of fit *S* are based on *F*^2^, conventional *R*-factors *R* are basedon *F*, with *F* set to zero for negative *F*^2^. The threshold expression of*F*^2^ > σ(*F*^2^) is used only for calculating *R*-factors(gt) *etc*. and isnot relevant to the choice of reflections for refinement. *R*-factors basedon *F*^2^ are statistically about twice as large as those based on *F*, and *R*-factors based on ALL data will be even larger.

### Fractional atomic coordinates and isotropic or equivalent isotropic displacement parameters (Å^2^)

**Table:**

	*x*	*y*	*z*	*U*_iso_*/*U*_eq_	
C1	0.3317 (3)	0.3973 (2)	0.5333 (4)	0.0380 (8)	
C2	0.2695 (3)	0.37563 (19)	0.6865 (4)	0.0354 (8)	
C3	0.3314 (3)	0.37812 (17)	0.8474 (6)	0.0512 (8)	
H3A	0.2903	0.3651	0.9499	0.061*	
C4	0.4498 (3)	0.3992 (2)	0.8561 (6)	0.0560 (10)	
H4A	0.4887	0.4012	0.9637	0.067*	
C5	0.5121 (3)	0.4175 (2)	0.7045 (6)	0.0602 (11)	
H5A	0.5931	0.4319	0.7101	0.072*	
C6	0.4544 (3)	0.4145 (2)	0.5448 (5)	0.0510 (10)	
H6A	0.4982	0.4240	0.4431	0.061*	
C7	0.1437 (3)	0.34815 (19)	0.6801 (4)	0.0399 (8)	
N8	0.0753 (2)	0.36544 (15)	0.5463 (3)	0.0378 (7)	
C9	−0.0510 (3)	0.3371 (2)	0.5463 (4)	0.0422 (9)	
H9A	−0.0956	0.3677	0.6353	0.051*	
H9B	−0.0549	0.2770	0.5733	0.051*	
C10	−0.1063 (3)	0.3534 (2)	0.3683 (4)	0.0407 (9)	
N11	−0.06106 (19)	0.44045 (15)	0.3205 (5)	0.0408 (6)	
H11A	−0.0946	0.4790	0.3925	0.049*	
H11B	−0.0844	0.4528	0.2107	0.049*	
V12	0.12859 (4)	0.45059 (3)	0.33674 (6)	0.03758 (15)	
O13	0.27910 (18)	0.39910 (13)	0.3758 (3)	0.0456 (6)	
O14	0.1359 (2)	0.45725 (14)	0.1236 (2)	0.0467 (6)	
O15	0.1372 (2)	0.54432 (13)	0.4218 (2)	0.0483 (6)	
C26	0.0950 (3)	0.29854 (18)	0.8337 (6)	0.0592 (9)	
H26A	0.0162	0.2776	0.8060	0.089*	
H26B	0.0905	0.3349	0.9343	0.089*	
H26C	0.1474	0.2516	0.8584	0.089*	
C27	−0.0620 (3)	0.2904 (2)	0.2305 (5)	0.0536 (9)	
H27A	0.0243	0.2886	0.2320	0.080*	
H27B	−0.0890	0.3081	0.1170	0.080*	
H27C	−0.0933	0.2349	0.2559	0.080*	
C28	−0.2434 (3)	0.3536 (2)	0.3770 (5)	0.0699 (12)	
H28A	−0.2697	0.3960	0.4587	0.105*	
H28B	−0.2713	0.2988	0.4143	0.105*	
H28C	−0.2756	0.3661	0.2633	0.105*	

### Atomic displacement parameters (Å^2^)

**Table:**

	*U*^11^	*U*^22^	*U*^33^	*U*^12^	*U*^13^	*U*^23^
C1	0.039 (2)	0.0260 (19)	0.049 (2)	0.0093 (17)	0.0054 (19)	0.0025 (17)
C2	0.041 (2)	0.0303 (19)	0.035 (2)	0.0077 (17)	0.0034 (17)	−0.0038 (16)
C3	0.064 (2)	0.0462 (19)	0.043 (2)	0.0081 (17)	0.007 (3)	0.005 (3)
C4	0.058 (3)	0.056 (2)	0.054 (3)	0.0082 (19)	−0.009 (3)	−0.016 (3)
C5	0.039 (2)	0.053 (3)	0.089 (3)	−0.0058 (19)	−0.002 (3)	−0.005 (2)
C6	0.050 (3)	0.051 (2)	0.052 (2)	0.001 (2)	0.009 (2)	0.003 (2)
C7	0.056 (2)	0.0311 (19)	0.033 (2)	0.0004 (19)	0.0127 (18)	−0.0038 (14)
N8	0.0384 (19)	0.0394 (17)	0.0355 (16)	0.0009 (14)	0.0112 (14)	−0.0026 (14)
C9	0.053 (3)	0.039 (2)	0.0346 (19)	−0.0049 (19)	0.0174 (18)	−0.0038 (17)
C10	0.038 (2)	0.0445 (19)	0.040 (2)	−0.0079 (17)	0.0074 (17)	−0.0095 (18)
N11	0.0423 (14)	0.0400 (14)	0.0400 (13)	0.0048 (13)	−0.0037 (18)	−0.0058 (18)
V12	0.0404 (3)	0.0357 (3)	0.0366 (3)	0.0011 (3)	0.0062 (4)	0.0047 (4)
O13	0.0417 (13)	0.0517 (14)	0.0434 (17)	0.0064 (10)	0.0104 (11)	0.0074 (12)
O14	0.0508 (14)	0.0579 (14)	0.0315 (11)	0.0065 (16)	0.0078 (11)	0.0070 (11)
O15	0.0559 (16)	0.0353 (13)	0.0538 (12)	−0.0029 (13)	0.0005 (11)	−0.0044 (11)
C26	0.082 (3)	0.0554 (19)	0.0399 (16)	−0.0127 (18)	0.015 (3)	0.005 (3)
C27	0.067 (2)	0.047 (2)	0.0469 (18)	−0.008 (2)	0.013 (2)	−0.007 (2)
C28	0.046 (2)	0.085 (3)	0.078 (3)	−0.017 (2)	0.004 (2)	−0.004 (3)

### Geometric parameters (Å, °)

**Table:**

C1—O13	1.339 (4)	C10—N11	1.504 (4)
C1—C6	1.393 (5)	C10—C28	1.527 (4)
C1—C2	1.402 (4)	C10—C27	1.529 (4)
C2—C3	1.410 (5)	N11—V12	2.118 (2)
C2—C7	1.465 (4)	N11—H11A	0.9000
C3—C4	1.359 (4)	N11—H11B	0.9000
C3—H3A	0.9300	V12—O15	1.6151 (19)
C4—C5	1.380 (5)	V12—O14	1.6352 (18)
C4—H4A	0.9300	V12—O13	1.883 (2)
C5—C6	1.380 (4)	C26—H26A	0.9600
C5—H5A	0.9300	C26—H26B	0.9600
C6—H6A	0.9300	C26—H26C	0.9600
C7—N8	1.304 (4)	C27—H27A	0.9600
C7—C26	1.510 (4)	C27—H27B	0.9600
N8—C9	1.473 (4)	C27—H27C	0.9600
N8—V12	2.171 (3)	C28—H28A	0.9600
C9—C10	1.515 (4)	C28—H28B	0.9600
C9—H9A	0.9700	C28—H28C	0.9600
C9—H9B	0.9700		
			
O13—C1—C6	118.7 (3)	C10—N11—V12	112.76 (17)
O13—C1—C2	122.7 (3)	C10—N11—H11A	109.0
C6—C1—C2	118.5 (3)	V12—N11—H11A	109.0
C1—C2—C3	118.7 (3)	C10—N11—H11B	109.0
C1—C2—C7	121.0 (3)	V12—N11—H11B	109.0
C3—C2—C7	120.2 (3)	H11A—N11—H11B	107.8
C4—C3—C2	121.5 (4)	O15—V12—O14	109.85 (10)
C4—C3—H3A	119.2	O15—V12—O13	106.06 (11)
C2—C3—H3A	119.2	O14—V12—O13	98.12 (10)
C3—C4—C5	119.7 (4)	O15—V12—N11	98.71 (11)
C3—C4—H4A	120.1	O14—V12—N11	89.77 (13)
C5—C4—H4A	120.1	O13—V12—N11	149.49 (10)
C6—C5—C4	120.1 (4)	O15—V12—N8	106.46 (9)
C6—C5—H5A	119.9	O14—V12—N8	142.05 (11)
C4—C5—H5A	119.9	O13—V12—N8	81.94 (9)
C5—C6—C1	121.2 (3)	N11—V12—N8	74.05 (12)
C5—C6—H6A	119.4	C1—O13—V12	122.65 (18)
C1—C6—H6A	119.4	C7—C26—H26A	109.5
N8—C7—C2	121.4 (3)	C7—C26—H26B	109.5
N8—C7—C26	120.6 (3)	H26A—C26—H26B	109.5
C2—C7—C26	118.0 (3)	C7—C26—H26C	109.5
C7—N8—C9	119.5 (3)	H26A—C26—H26C	109.5
C7—N8—V12	123.3 (2)	H26B—C26—H26C	109.5
C9—N8—V12	116.56 (19)	C10—C27—H27A	109.5
N8—C9—C10	109.6 (2)	C10—C27—H27B	109.5
N8—C9—H9A	109.8	H27A—C27—H27B	109.5
C10—C9—H9A	109.8	C10—C27—H27C	109.5
N8—C9—H9B	109.8	H27A—C27—H27C	109.5
C10—C9—H9B	109.8	H27B—C27—H27C	109.5
H9A—C9—H9B	108.2	C10—C28—H28A	109.5
N11—C10—C9	103.7 (3)	C10—C28—H28B	109.5
N11—C10—C28	110.0 (2)	H28A—C28—H28B	109.5
C9—C10—C28	111.5 (3)	C10—C28—H28C	109.5
N11—C10—C27	108.4 (3)	H28A—C28—H28C	109.5
C9—C10—C27	112.2 (3)	H28B—C28—H28C	109.5
C28—C10—C27	110.7 (3)		
			
O13—C1—C2—C3	−178.0 (3)	N8—C9—C10—C27	74.2 (3)
C6—C1—C2—C3	4.6 (4)	C9—C10—N11—V12	53.3 (3)
O13—C1—C2—C7	4.0 (4)	C28—C10—N11—V12	172.7 (2)
C6—C1—C2—C7	−173.4 (3)	C27—C10—N11—V12	−66.2 (3)
C1—C2—C3—C4	−1.5 (4)	C10—N11—V12—O15	−139.7 (2)
C7—C2—C3—C4	176.6 (3)	C10—N11—V12—O14	110.2 (3)
C2—C3—C4—C5	−0.9 (5)	C10—N11—V12—O13	4.5 (4)
C3—C4—C5—C6	0.0 (5)	C10—N11—V12—N8	−35.0 (2)
C4—C5—C6—C1	3.4 (6)	C7—N8—V12—O15	−66.8 (3)
O13—C1—C6—C5	176.9 (3)	C9—N8—V12—O15	103.9 (2)
C2—C1—C6—C5	−5.6 (5)	C7—N8—V12—O14	130.5 (2)
C1—C2—C7—N8	−20.0 (4)	C9—N8—V12—O14	−58.7 (3)
C3—C2—C7—N8	162.0 (3)	C7—N8—V12—O13	37.6 (2)
C1—C2—C7—C26	159.8 (3)	C9—N8—V12—O13	−151.6 (2)
C3—C2—C7—C26	−18.2 (4)	C7—N8—V12—N11	−161.4 (3)
C2—C7—N8—C9	−179.5 (3)	C9—N8—V12—N11	9.3 (2)
C26—C7—N8—C9	0.8 (4)	C6—C1—O13—V12	−136.7 (2)
C2—C7—N8—V12	−9.0 (4)	C2—C1—O13—V12	45.9 (4)
C26—C7—N8—V12	171.2 (2)	O15—V12—O13—C1	50.2 (2)
C7—N8—C9—C10	−172.0 (3)	O14—V12—O13—C1	163.6 (2)
V12—N8—C9—C10	16.9 (3)	N11—V12—O13—C1	−92.8 (3)
N8—C9—C10—N11	−42.6 (3)	N8—V12—O13—C1	−54.7 (2)
N8—C9—C10—C28	−161.0 (3)		

### Hydrogen-bond geometry (Å, °)

**Table:**

*D*—H···*A*	*D*—H	H···*A*	*D*···*A*	*D*—H···*A*
N11—H11A···O14^i^	0.90	2.08	2.942 (4)	159
N11—H11B···O15^ii^	0.90	2.29	3.173 (4)	168
C26—H26B···O14^iii^	0.96	2.46	3.370 (4)	158

Symmetry codes:  (i) −*x*, −*y*+1, *z*+1/2; (ii) −*x*, −*y*+1, *z*−1/2; (iii) *x*, *y*, *z*+1.
